# Radiation Hardness of 4H-SiC P-N Junction UV Photo-Detector

**DOI:** 10.3390/ma15010264

**Published:** 2021-12-30

**Authors:** Antonella Sciuto, Lucia Calcagno, Salvatore Di Franco, Domenico Pellegrino, Lorenzo Maurizio Selgi, Giuseppe D’Arrigo

**Affiliations:** 1 CNR-Institute for Microelectronic and Microsystems, VIII Strada No. 5, 95121 Catania, Italy; salvatore.difranco@imm.cnr.it (S.D.F.); domenico.pellegrino@ct.infn.it (D.P.); giuseppe.darrigo@imm.cnr.it (G.D.); 2 Department of Physics and Astronomy, University of Catania, 95123 Catania, Italy; lucia.calcagno@ct.infn.it; 3 STMicroelectronics, Str.le Primosole 50, 95121 Catania, Italy; lorenzo.selgi@st.com

**Keywords:** silicon carbide, p-n diode, UV photo-detector, defects, ion irradiation, radiation hardness, optical responsivity, ion irradiation

## Abstract

4H-SiC based p-n junction UV photo-detectors were irradiated with 600 keV He^+^ in the fluence range of 5 × 10^11^ ÷ 5 × 10^14^ ion/cm^2^ in order to investigate their radiation hardness. The effects of irradiation on the electro-optical performance were monitored in dark condition and in the UV (200 ÷ 400 nm) range, as well as in the visible region confirming the typical visible blindness of unirradiated and irradiated SiC photo-sensors. A decrease of UV optical responsivity occurred after irradiation and two fluence regimes were identified. At low fluence (<10^13^ ions/cm^2^), a considerable reduction of optical responsivity (of about 50%) was measured despite the absence of relevant dark current changes. The presence of irradiation induced point defects and then the reduction of photo-generated charge lifetime are responsible for a reduction of the charge collection efficiency and then of the relevant optical response reduction: point defects act as recombination centers for the photo-generated charges, which recombine during the drift/diffusion toward the electrodes. At higher irradiation fluence, the optical responsivity is strongly reduced due to the formation of complex defects. The threshold between low and high fluence is about 100 kGy, confirming the radiation hardness of SiC photo-sensors.

## 1. Introduction

Silicon Carbide (SiC) receives increasing attention thanks to its interesting physics and electronic properties, for instance, large band-gap, high thermal conductivity, high breakdown field, and high electron mobility [1], and it is considered an ideal material for the fabrication of high power devices and high temperature electronics [2,3]. Other properties such as the transparency to visible light, the UV-photons absorption, the radiation hardness, and the biocompatibility make this material attractive for alternative applications, for instance, biomedical sensors and X-ray, UV, and charged particles detectors [4,5,6,7]. Applications involve visible light and hostile environment such as, for example, the monitoring of laser generated plasma or of flames in industrial apparatus found large prominence [8,9].

In the last years, great attention has been paid to the study of radiation hardness of particle detectors based on Schottky diodes and on p-n junctions by studying the performance changes after irradiation with gamma rays, neutrons, protons, high energy ions, and intense photon flux [10,11,12,13,14,15,16,17]. Recently, we have shown [18] that the defects introduced by He irradiation in a 4H-SiC based p-n junctions can be exploited to fabricate photon sources operating in the NIR and we also published a wide study on the electrical performance of ion irradiated 4H-SiC p-n junctions correlating their evolution with irradiation defects [19]. As it is well known, p-n junction is an essential element of more complicated devices such as MOSFET or avalanche photodiode, widely investigated in the literature for extreme environment UV monitoring applications [20,21,22]. In this field, electro-optical performance changes due to cosmic rays and, more generally, to particle irradiation need to be managed. Nevertheless, the literature works on the irradiation effects on the UV photo-response are few in the case of the Schottky type device [12,13,14] and to our knowledge, are absent in the case of p-n junction based devices. 

In the present work, we studied and discuss on the effects of He irradiation on p-n junction photo-detectors. In particular, the electro-optical performance of the device was monitored as a function of irradiation fluence and changes in the optical measurements were correlated to the irradiation defects. The irradiation dose absorbed by our samples was estimated adopting the ion deposited energy calculated by SRIM and irradiation fluences were fixed to span a wide range of absorbed dose, from few kGy to MGy, emulating an irradiation regime of different applications [23,24].

## 2. Materials and Methods

Devices were fabricated in the CNR-IMM Catania clean-room facilities using a 1.2 µm thick n-type (5 × 10^17^ donor/cm^3^) 4H-SiC epitaxial layer (grown by LPE – Epitaxial Technology Center, Catania, Italy) on a n-type heavily doped (1 × 10^19^ donor/cm^3^) 4H-SiC substrate. 

The p shallow regions were obtained in the n-type epi-layer through a high dose and low energy Al^+^ multiple implantation process. A dedicated high-temperature thermal annealing process at 1700 °C for 30 min in Ar ambient was adopted to activate the dopant and to recover the implantation damage. This annealing was carried out by using a graphitic capping layer to avoid surface step bunching [25,26,27].

**Figure 1:**
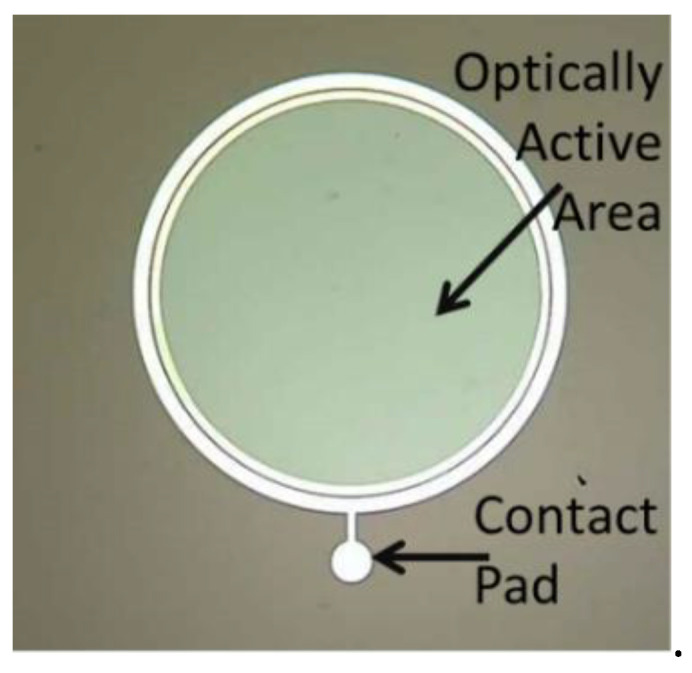
Picture of one of fabricated devices: anodic Ni_2_Si top electrode has a circular crown geometry; it is not visible in the picture being surmounted by the Al metal contact and pad.

Previous works [18,25,26] show that the p region, obtained as described in previous lines, has a depth of about 175 nm and an activated dopant concentration of about 5 × 10^18^ /cm^3^. Further details concerning the devices fabrication are reported in [26]. The Ohmic contacts on the n^+^ back substrate and on the p top surface were formed by sputtering a 200 nm and a 100 nm thick Ni films, followed by rapid annealing processes at 1000 °C and at 900 °C, respectively, in N_2_ ambient to form Ni_2_Si electrodes [28]. The devices are laterally surrounded by a 1.6 μm thick TEOS (tetraethylorthosilicate) ring and aluminum contact pads were deposited on the front side for the anode region contact. A picture of one of devices studied in this paper is reported in Figure 1: the anodic Ni_2_Si top electrode has a circular crown geometry and is surmounted by the Al metal contact and pad, both visible in Figure 1.

The devices have an optically active area (OAA) about 0.8 mm^2^ wide, directly exposed to the incident optical radiation thanks to the anodic electrode crown geometry [29].

The p-n diodes were successively irradiated at room temperature with 600 keV He^+^ ions at different fluences in the range 5 × 10^11^–5 × 10^14^ ion/cm^2^. The ion energy was enough to entirely cross the 1.2 μm thick n-type epilayer. Helium irradiation allows inducing damage without doping effects and SRIM simulation was performed to evaluate the defects profile and to calculate the displacement per atom (dpa) [30]. In particular, we calculated that the dpa in the case of 5 × 10^14^ ion/cm^2^ irradiation fluence is about 0.02, then very low with respect to the amorphisation threshold of 0.55 reported in literature for our semiconductor [31]. Moreover, from SRIM simulations we estimated the absorbed dose of our devices is in the range 4.7 × 10^3^–4.7 × 10^6^ Gy (or 470 krad–470 Mrad) [23,24]. 

Devices electrical characterization in static condition has been carried out using a Keithley 2636 Source Meter Unit. The optical characterizations have been carried using a Xenon lamp assembly as the luminous source, a CVI/Digikrom DK240 monochromator, a 100 μm diameter core optical fiber provided with focusing system and a commercial Ophir-Optronics power meter used to calibrate the apparatus. An automated procedure was used to calculate the optical Responsivity during the measurements: device photocurrent was measured spanning the wavelength interval of interest and Responsivity was calculated using the optical power calibration curve acquired at each measurements run [6]. The dark current was first measured at each reverse bias value used for the characterization and then subtracted from the measured photocurrent values. 

## 3. Results and Discussion

As known, photo-diodes can operate in photovoltaic mode (zero applied voltage) or in reverse polarization condition. In general, the photocurrent (*I_ph_*) can be expressed as the sum of two contributions: a contribution (*I_drift_*) due to the drift of charge carriers photo-generated inside the junction depleted region (W) and moving under the effect of the electric field; and a second contribution (*I_diff_*) due to the charge carriers photo-generated outside the depleted region, but at a distance from it less than the diffusion length and then able to reach the depleted region by diffusion:

$$I_{ph}=I_{drift}+I_{diff}$$

In the presence of external reverse voltage applied to the detector, the extension of depleted region W is almost proportional to the square root of the applied voltage (V) and thus also the drift current (being correlated to the depleted region extension) [32,33]. On the other side, the diffusion photocurrent component, more relevant in photovoltaic operation mode, is particularly affected by the presence of defects that reduce the average lifetime and therefore the diffusion length of charge carriers, as discussed in the next pages. In any case, low values of dark (leakage) current are crucial for the optimal photo-detector operation, in order to ensure an elevated signal to noise ratio. Reverse I–V characteristic in dark condition is a crucial parameter that we monitored at room temperature, obtaining in unirradiated devices dark current a density lower than 25 nA/cm^2^ up to 20 V reverse polarization. Measurements were limited to 20 V bias value being sufficient to monitor the electro-optical performance and the effects of damage in the junction region while avoiding lateral leakage current taking place due to the absence of device lateral guard ring.

**Figure 2:**
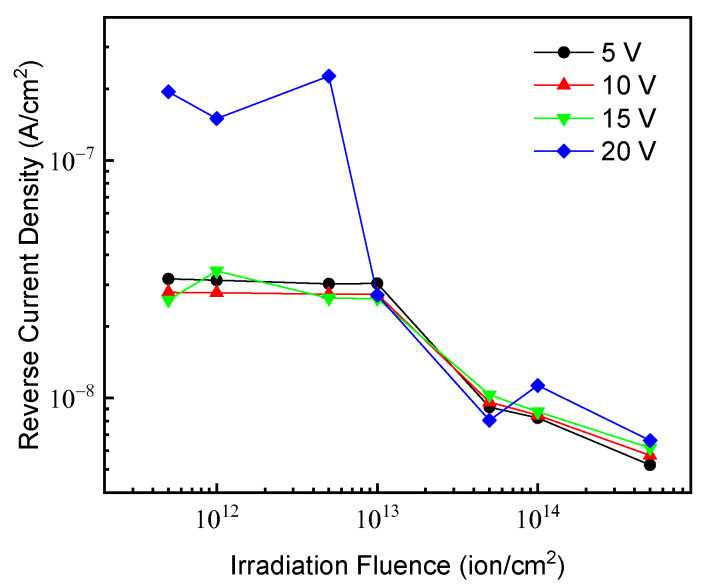
Dark current density values at 5, 10, 15, and 20 V reverse polarization of photodiodes after irradiation at different He ions fluences.

The reverse dark current measurements were carried out also in the irradiated devices. To examine the irradiation effects the values of the current obtained at different values of the reverse voltage versus the irradiation fluences are resumed in Figure 2. With respect to the unirradiated diode (not shown in figure and having a current density <25 nA/cm^2^ in the entirely explored voltage range), the irradiated devices with fluence up to 1 × 10^13^ ion/cm^2^ show a reasonable increase of the dark current with bias in particular at high values (20 V) of the reverse voltage. Instead, a relevant reduction of the reverse current with respect to the unirradiated one is observable in the devices irradiated at higher ions fluences (>1 × 10^13^ ion/cm^2^). 

This effect was already observed in similar devices irradiated with 700 keV He ions and was correlated to the reduction of carrier concentration with irradiation dose. In fact, point defects introduced by irradiation exhibit acceptor-like behavior and compensation effects take place inducing a decrease of carrier concentration [19,34].

**Figure 3:**
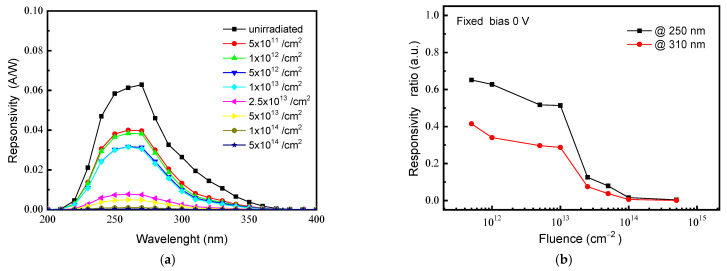
Responsivity (R) spectra of unirradiated and irradiated diodes in the UV range measured at 0 V in the wavelength region 200 ÷ 400 nm (**a**); responsivity ratio (R) after irradiation and its value before irradiation) versus irradiation fluence for the two wavelengths of 250 and 310 nm (**b**).

In addition to the electrical performances, a crucial parameter of photo-detectors is the electro-optical response. Responsivity (R) spectra of unirradiated and irradiated diodes were measured in the UV and in the Visible range; in Figure 3a are reported the R spectra measured at 0 V in the wavelength region 200–400 nm; the response of all devices at longer wavelengths (not shown for brevity) is almost null, confirming the visible blindness (typical of 4H-SiC photo-sensors and widely discussed in our previous works [6,26,29]) and then excluding the presence of optically active defects, also in the irradiated devices. The unirradiated diode exhibits a maximum response of ~0.063 A/W at 260 nm that reduces by 90% for wavelengths below ~230 nm and above ~340 nm. Ion irradiation induces an overall reduction of photo-response, although the absence of detriment effects on the dark current (see Figure 2) excludes the introduction of carrier generation sites in irradiated devices. At fluence of 1 × 10^13^ ion/cm^2^, the R reduction is not drastic, being the peak value of ~0.03 A/W (about halved with respect to the unirradiated starting value); instead, at 2.5 × 10^13^ ion/cm^2^ irradiation fluence, the R peak crashes to the ~0.008 A/W value. The relevant optical response decrease could be related to a reduction of the charge collection efficiency: point defects act as recombination centers (reduction of charge carrier lifetime) for the photo-generated charges, which recombine during their path toward the electrodes.

From the R spectra of Figure 3a, we extracted and plotted in Figure 3b the ratio of responsivity after and before irradiation, for the two wavelengths of 250 nm and 310 nm. These two wavelengths (250 nm and 310 nm) have been fixed because their experimental penetration depths in 4H-SiC are well known in literature and because they are sufficiently distant each other to represent two different spectrum regions, the first one near the detector response peak and the second one in a low, but not null, response spectrum region [35,36]. The results reported in Figure 3b evidence two effects: (1) a fluence dependence of the response which is similar for both wavelengths with a slow decrease at low fluence (≤1 × 10^13^ ion/cm^2^) and a steep decrease at higher fluences; (2) a wavelength dependence at low fluence, as the responsivity reduction is about 0.5 at 250 nm and 0.3 at 310 nm for the 1 × 10^13^ ion/cm^2^ fluence.

The 1 × 10^13^ ion/cm^2^ fluence is a kind of threshold between a low irradiation regime, were an overall deterioration of the photo-detector performance are observed, and a high irradiation regime, were the photo-detection abilities are almost totally inflicted. As mentioned in the previous page, in reference 19, trough high temperatures, electrical measurements, and DLTS analysis, we deduced that at low fluence (≤1 × 10^13^ ions/cm^2^) point defects, such as Z_1/2_, RD_1/2_, and EH_6/7_, are produced whose concentration increases with fluence and leads to a decrease of free carrier concentration and lifetime. At high fluence (>1 × 10^13^ ions/cm^2^), it occurs a rearrangement of primary defects that agglomerate creating complex defects responsible for the extra-current in forward polarization and for the high resistivity regions formation (growth of series resistance). In order to investigate on nature of these defects, advanced structural characterizations were performed by transmission electron microscopy (TEM) on the higher fluence irradiated device, but they have not evidenced the presence of observable defects confirming that only point defects or very small agglomerates (clusters) are produced in our device [19]. 

The corresponding threshold of absorbed dose, evaluated as discussed in the previous page, is of ~90 kGy (100 kGy).

Instead, the different value of responsivity ratio measured at low fluence (≤the 1 × 10^13^ ion/cm^2^) for the two wavelengths of 250 nm and 310 nm reported in Figure 3b can be explained by considering two effects: (i) the irradiation damage distribution and (ii) the different photons penetration depths in the semiconductor.

**Figure 4:**
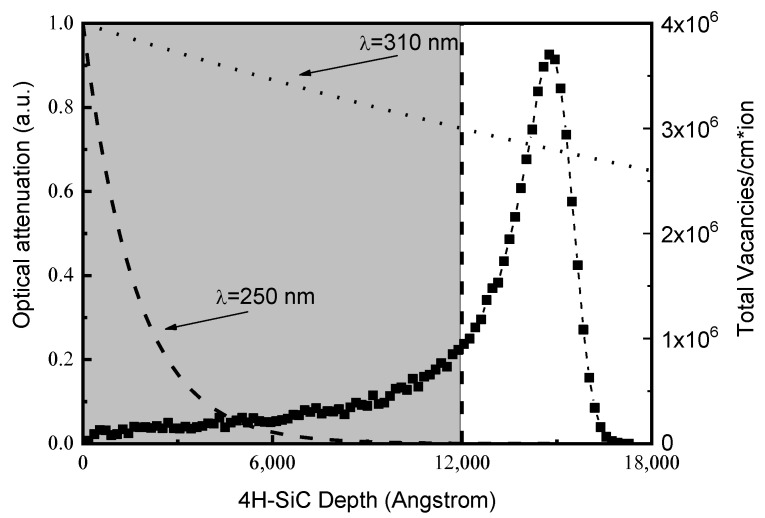
Total vacancies distribution induced by 600 keV He^+^ ion in SiC evaluated by SRIM code (right axis); the optical attenuation of impinging radiation inside the semiconductor for the 250 nm and 310 nm radiation wavelengths, calculated using the literature data of the extinction coefficient [21] and the well-known Lambert–Beer law (left axis).

Concerning the damage, as yet said, the total vacancies distribution induced by 600 keV He^+^ ion in SiC is evaluated by SRIM simulation [23], and is reported in Figure 4, referred to the right axis. The total vacancies profile exhibits a maximum at a depth of 1.5 μm, deeper than the epitaxial layer; the vacancies number changes through the epilayer from about 1.6 × 10^5^ vacancies/cm/ion at ~200 nm from the surface, in correspondence of the p-n junction (see [19,23]), to about 4 × 10^6^ vacancies/cm/ion at its the peak. In the same Figure 4, referred to the left axis, is also reported the optical attenuation I/I_0_, with I_0_ impinging radiation intensity and I intensity transmitted through the semiconductor. The attenuation I/I_0_ versus the silicon carbide traversed thickness was calculated for 250 nm and 310 nm wavelengths using the literature extinction coefficient values and the well-known Lambert–Beer law [35,36,37]. As observable in Figure 4, the 250 nm radiation is absorbed in a surface layer about 0.5 μm thick (left axis), where the vacancies number is in the range of 2 × 10^5^ vacancies/cm/ion (right axis); instead, only the 20% of the 310 nm radiation is absorbed in the epilayer, penetrating well beyond the 1.2 μm tick active layer (left axis), where the vacancies concentration reaches value of 9 × 10^5^ vacancies/cm/ion (right axis). It is evident how the most penetrating radiation (longer wavelength) generates the photo-carriers in a region with greater concentration of defects. This explains why reduced values of Responsivity are measured at long wavelengths, already at low irradiation fluences (see Figure 3b).

As described in previous pages, the optical response depends on the reverse bias through the depleted region extension (W); complete responsivity spectra were then measured for all tested devices for different reverse biases and, as expected, a relevant increase of the optical response is observed increasing the device polarization, both in unirradiated and in irradiated detectors. Between irradiated samples, the 1 *×* 10^13^ ion/cm^2^ fluence (damage threshold) was selected for our successive discussion.

**Figure 5:**
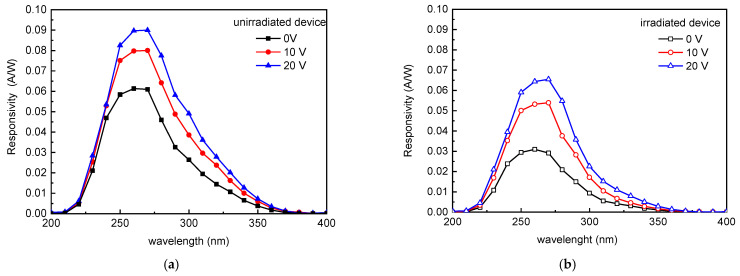
Responsivity spectra of unirradiated device (**a**) and of device irradiated at 1 × 10^13^ ion/cm^2^ fluence (**b**) measured in photovoltaic configuration (@ 0 V) and in reverse polarization condition (@ 10 V and @ 20 V).

The complete R spectra obtained at 0, 10 and 20 V device reverse biases are plotted in Figure 5: the spectra of the unirradiated diode are plotted in Figure 5a with full squares, circles, and triangles for the different examined biases; those of irradiated device, with the same criterion, are plotted with empty symbols in Figure 5b. 

The spectra keep their bell shape, asymmetrical with respect to the peak, as the voltage increases (in absolute value): in the unirradiated device, the R peak, that is ~0.06 A/W @ 0 V, exhibits a relevant increase joining ~0.09 A/W at 20 V (reverse bias); a similar trend is observed in the irradiated device where the R value changes from 0.03 A/W to 0.068 A/W.

**Figure 6:**
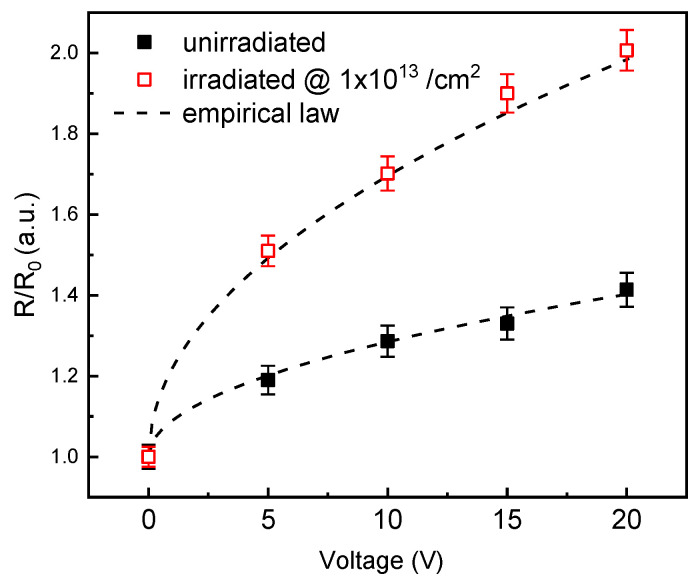
Responsivity ratio (*R*⁄*R*_0_) versus applied voltage of unirradiated (full symbols) and 10^13^ ion/cm^2^ irradiated devices (empty symbols). The plots are relative to the peak wavelength.

In order to discuss the effect of photo-detector bias on the responsivity, we calculated (from data of Figure 5), the ratio (*R*/*R*_0_) between the optical device response in presence of applied voltage, and the response at 0 V, at the peak (260 nm) both in unirradiated and irradiated devices. The obtained values versus applied device voltage are plotted in Figure 6.

As observable in Figure 6, the response increment passing from 0 V to 20 V is about 1.3 in the unirradiated diode (full symbols in figure); in the same voltage range, a greater growth rate is obtained in the irradiated device (empty symbols) with a doubling of the response. 

The optical response and then the *R*/*R*_0_ ratio versus bias reported in Figure 6, follows the empirical low

RR0=1+PV

where *V* is the applied device voltage [32]. The pre-factor *P* is calculated superimposing to the experimental data the aforementioned law and values of 0.09 V*^−^*^1/2^ and 0.22 V*^−^*^1/2^ are obtained for the unirradiated and irradiated devices, respectively. 

As discussed in previous pages, the photocurrent is the sum of the drift and of the diffusion components. This second is independent from the bias and is correlated to the diffusion length (charge carrier lifetime) which is certainly reduced in the irradiated devices due to the presence and/or the increase of concentration of defects in the semiconductor. Concerning the drift component, it increases with bias and the higher value of the pre-factor P in the irradiated device is in agreement with the reduction of free carrier concentration of the epilayer: a wider depleted junction region is obtained due to the dopant deactivation discussed in previous pages; as consequence a more relevant effect of bias on the optical Response of irradiated devices is observed.

The obtained results indicate the applied reverse bias increase can compensate the irradiation induced optical response detriment. Taking into account the dopant deactivation effects, it is then possible do predict the changes in the optical device response and to adopt opportune device polarization to compensate the irradiation induced response detriment: as observable in Figure 5a,b the response at 20 V of irradiated device (absorbed dose ~90 kGy) approaches the response of unirradiated device at 0 V. 

## 4. Conclusions

This paper is focused on the study of irradiation effects on the electro-optical performance of p-n junction UV detectors. To this purpose, devices fabricated by Al implantation on 4H-SiC n-type epitaxial layer were subjected to He irradiation at 600 keV energy and at fluences in a wide range corresponding to an absorbed dose between few kGy and few MGy. The devices optical response was studied before and after irradiation in the UV and in the Visible range: first of all, optical measurements confirmed the visible blindness of photo-diodes also after irradiation; secondly, the evolution of the responsivity in the UV was investigated, correlating the observed changes with the irradiation defects. In particular, our experiment evidenced the 1 × 10^13^ ion/cm^2^ fluence, and as consequence, the absorbed dose of ~90 kGy is a threshold between a low dose irradiation regime, where an overall not drastic deterioration of the photo-detector performance is observed, and a high irradiation regime, where the photo-detection abilities are almost totally inflicted. 

The study of the optical response at different wavelengths and different reverse device polarizations and their correlation with the UV photons penetration depth and with the irradiation defects distribution allowed to speculate the reduction of the charge collection efficiency and, in particular, of the carrier lifetime affecting more penetrating wavelength and compensable acting on device biasing voltage.

The actual study is of crucial importance for all applications involving UV detectors subjected to irradiation and highlights how the response spectrum of the SiC photo-devices is affected in different way at different wavelengths. Knowledge of the operating environment of the device in terms of absorbed dose by the detector is essential to predict the alteration of the optical response of the exposed device itself. The use of the results obtained in this work, associated to literature results on irradiation defects, constitutes the starting point for the development of device simulation procedures predicting the evolution of electro-optical performance.

## Data Availability

The data presented in this study are available on request from the corresponding author.
